# Unraveling the Interplay between Quantum Transport and Geometrical Conformations in Monocyclic Hydrocarbons’ Molecular Junctions

**DOI:** 10.1021/acs.jpcc.3c05393

**Published:** 2023-11-27

**Authors:** A. Martinez-Garcia, T. de Ara, L. Pastor-Amat, C. Untiedt, E. B. Lombardi, W. Dednam, C. Sabater

**Affiliations:** † Departamento de Física Aplicada and Instituto Universitario de Materiales de Alicante (IUMA), Universidad de Alicante, Campus de San Vicente del Raspeig, Alicante E-03690, Spain; ‡ Department of Physics, Florida Science Campus, University of South Africa, Florida Park, Johannesburg 1710, South Africa

## Abstract

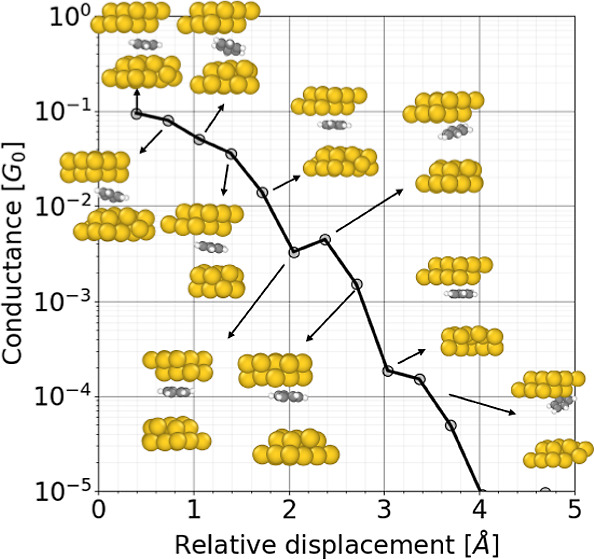

In the field of molecular electronics, especially in quantum transport experiments, determining the geometrical configurations of a single molecule trapped between two electrodes can be challenging. To address this challenge, we employed a combination of molecular dynamics (MD) simulations and electronic transport calculations based on density functional theory to determine the molecular orientation in our break-junction experiments under ambient conditions. The molecules used in this study are common solvents used in molecular electronics, such as benzene, toluene (aromatic), and cyclohexane (aliphatic). Furthermore, we introduced a novel criterion based on the normal vector of the surface formed by the cavity of these ring-shaped monocyclic hydrocarbon molecules to clearly define the orientation of the molecules with respect to the electrodes. By comparing the results obtained through MD simulations and density functional theory with experimental data, we observed that both are in good agreement. This agreement helps us to uncover the different geometrical configurations that these molecules adopt in break-junction experiments. This approach can significantly improve our understanding of molecular electronics, especially when using more complex cyclic hydrocarbons.

## Introduction

All the instruments and techniques used to measure the electronic transport of atomic-sized contacts were developed over more than three decades ago.^1,2^ At the beginning of that period, the electronic transport of atomic contacts made of metals,^3^ semimetals,^4^ and superconductors^5^ was studied, leading to an immediate interest in molecular junctions. The molecular electronics field initially considered very simple molecules like diatomic hydrogen and deuterium,^6^ which paved the way for obtaining knowledge from an experimental point of view. For various reasons and based on attempts at trying to identify exotic properties in the electronic transport field, innumerable types of different molecules have been studied.^7−19^ The creation of a molecular junction basically depends on its physical state and the method of deposition of the molecules, which can be grouped into three categories. The first one corresponds to the gas phase molecules, which are associated with the unique possibility of blowing the gas close to the electrodes. The second group comprises molecules in the solid state that can be deposited by thermal evaporation onto the junctions.^20^ The final group is composed of molecules that can be delivered in solution that need to wait for the solvent to evaporate before capturing the target molecules.

It is still widely believed that the solvent disappears or has a negligible effect on electronic transport after evaporation.^21^ However, recent works^19^ have demonstrated that the solvent’s presence is indeed detectable via scanning tunneling microscopy (STM) topography and STM-break junction (BJ) experiments. In our previous published experimental results,^19^ we identified the molecular electronic signature of the aromatic (benzene and toluene) and aliphatic (cyclohexane) molecules.^22,23^ Although studies have been conducted on the orientation of aromatic solvents with methyl anchoring groups, such as 1,3,5-trimethylbenzene,^24,25^ there have been no systematic studies exploring the relationship between the electronic transport and the geometric orientation for solvents without anchoring groups, such as benzene, toluene, or cyclohexane.

Recent works based on atomically precise binding conformations^26^ have inspired us to clearly identify the relationship between electronic transport and molecular orientation by using classical molecular dynamics (CMD) simulations and density functional theory (DFT) electronic transport calculations.^19^ Based on our simulations, we have classified the final contact for benzene, cyclohexane, and toluene. Furthermore, calculations of the electronic transport were performed using DFT for several scenarios provided by CMD. Finally, we compare our simulations and calculations with electronic transport experiments. This comparison helps us to unmask the relationship between the conductance and the orientation of molecules between the gold electrodes.

## Materials and Methods

### Identifying the Orientation of the Cyclic Molecules

**Figure 1:**
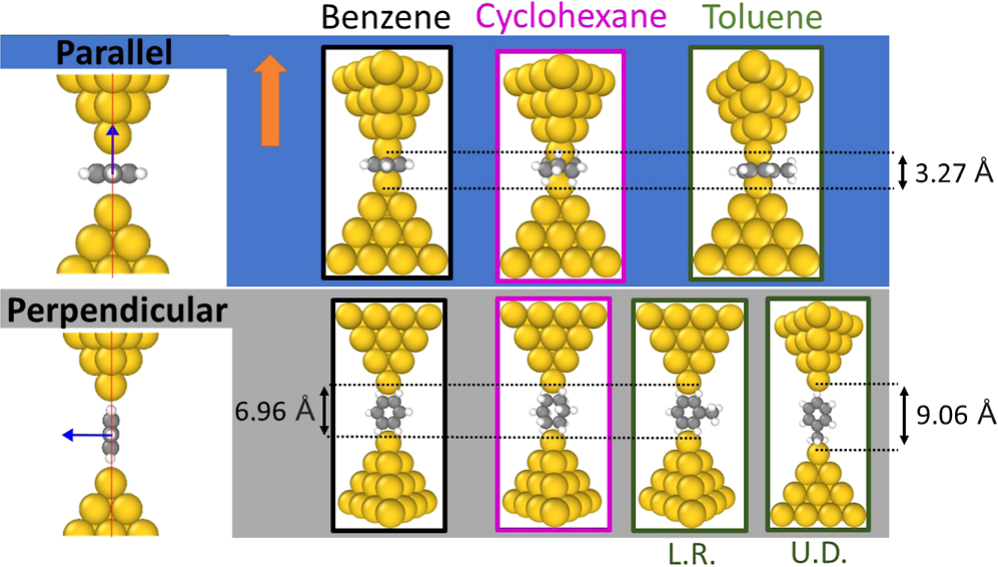
Illustration of “parallel” and “perpendicular” configurations of benzene molecules between two gold electrodes. The blue rectangle next to it shows the initial structures of the benzene, cyclohexane, and toluene junctions for one of the two configurations studied in this work. The starting distance between the two gold apex atoms of the tips in the “parallel” configuration is depicted with dashed lines and is 3.27 Å. Three of the “perpendicular” configurations have a starting distance between the gold tip apex atoms, indicated by dashed lines, of 6.96 Å. In the fourth “perpendicular” configuration, the toluene molecule with the methyl group attached to the upper electrode has gold tip atoms that start apart 9.06 Å. The “perpendicular” configuration of the toluene is labeled as L.R. and U.D., which indicates if the methyl group is located left–right or up–down.

From a mathematical point of view, the normal vector is the simplest way to identify a plane. Based on the fact that aromatic solvents (such as benzene and toluene) and aliphatic solvents (such as cyclohexane) analyzed in our article are monocyclic in nature, we use their internal cavity as the orientation plane. Its normal vector is used to identify if we are in parallel or in a perpendicular configuration with respect to the alignment vector defined by the electrodes. In the illustrations on the leftmost side of Figure 1, blue arrows indicate the normal vector to the plane defined by the cavity of the benzene molecule. The red line indicates the direction of the vector defined by the alignment of the electrodes, in this case, dominated by the center of the upper and bottom apex Au atoms of the leads. As a criterion, we have defined the parallel configuration when the normal vector forms an angle θ in the range of 0 to 15° with the alignment of the electrodes. On the other hand, we consider the “perpendicular” configuration when the angle θ is between 16° and 90°, with the extreme case being θ = 90°.

### Calculations of Conductance Based on DFT and CMD Simulations

To calculate the conductance, we employed DFT combined with the nonequilibrium Green’s function (NEGF) approach to quantum scattering.^27^ The electronic transport calculations were performed using the well-established code ANT.GAUSSIAN.^28−30^ This code is built on top of and interfaces with GAUSSIAN09. To ensure the high quality^31^ of our calculations, we used the LANL2DZ basis set^32^ for the atoms of the molecules and a few of the adjacent metal layers on either side of the molecules, while the outer metal layers were described by the smaller tight-binding-like CRENBS basis set.^33^ Additionally, we have decided to use the HSE06 functional, which remains the standard DFT approach for representing frontier-orbital levels of metal–organic systems accurately.^34−37^ This “range-corrected” functional achieves a balance between long–range interactions (as observed in metals) and local interactions (present in molecules or other systems with strong local interactions), as required for the systems studied in this work. It is important to note that using this functional incurs a higher computational cost in terms of time; however, for other studies where the computational cost could be a problem, we have compared HSE06 with B3LYP and BLYP, as shown in Figure S2.

In this work, we calculated the electronic transport in two scenarios: First, for idealized initial test structures such as those shown in Figure 1 and second for electrodes stretched by CMD simulations as described below. For each of these scenarios, the DFT quantum transport methodology remained the same.

A simulation based on CMD essentially solves Newton’s second law in order to obtain the trajectories of all the atoms involved in the phenomena it is meant to describe.^38,39^

To simulate the rupture–formation cycles of the gold nanowire with the target molecules, we used the LAMMPS code^40−42^ with a reactive force-field (ReaxFF) potential,^43,44^ which reproduces the single-molecule junctions^45,46^ and mechanical, catalytic behavior of metal–organic compounds.^47,48^

The simulated nanowire is oriented along the (001) crystallographic direction and has been created with an area of narrower cross-section (≈17 × 17 × 25 Å^3^) where rupture will take place.^49^ In this area of constriction, we place four molecules on each of the four square crystallographic facets of the gold nanowire (see the “Input” panels in Figure 3); hence, 16 molecules are deposited in total for each initial condition. This process is repeated for each of the substances: benzene, cyclohexane, or toluene in turn. In other words, the simulation is conducted separately for each of these three substances. To accurately replicate the experimental conditions, the simulations were conducted at a constant temperature of 300 K in an *NVT* canonical ensemble using a Nosé–Hoover thermostat. The simulations were performed with a time step of 1 fs. Snapshots of the atomic configuration were saved every 5000 steps. The pulling and pushing velocities were set to 0.00033 nm/fs, and the number of steps to form and destroy the contact in the continuous cycles was 224,000 to form the junction and 220,000 to break it. During the simulations, the internal motion of the atoms in the outermost three upper layers and three bottom layers was kept frozen.

**Figure 2:**
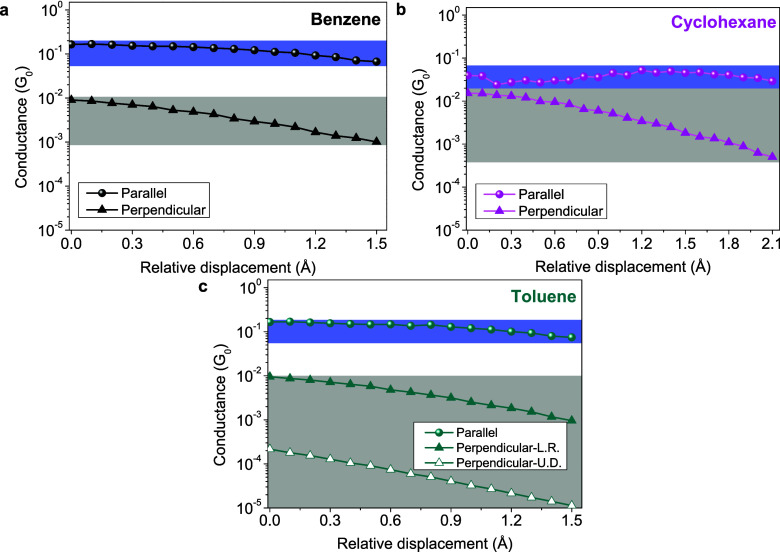
Calculated electronic transport vs relative displacement of the initial structures of benzene, cyclohexane, and toluene, labeled as a, b, and c. Colored markers represent the calculated values of benzene (black), cyclohexane (pink), and toluene (green).

**Figure 3:**
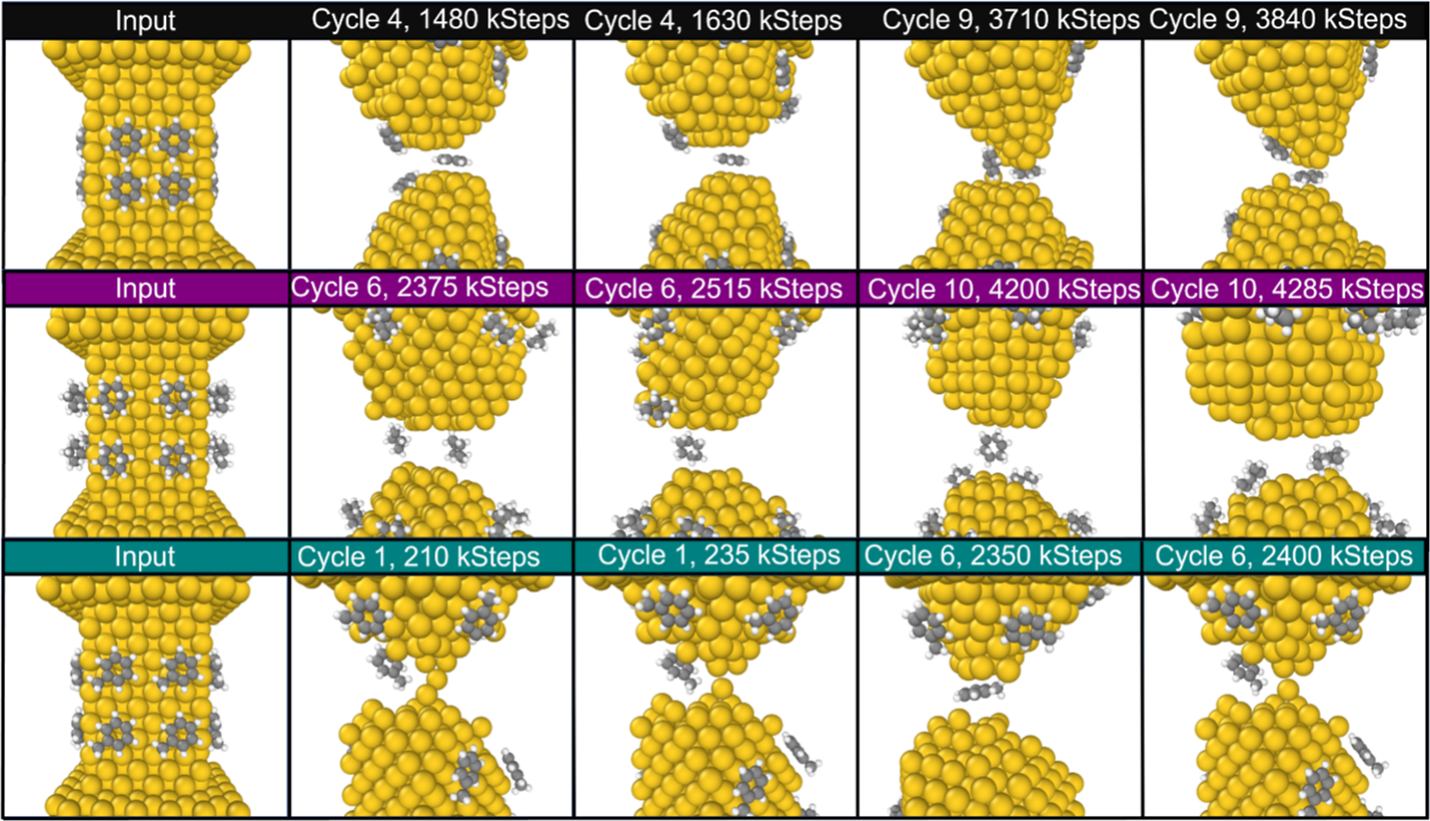
Snapshots of the simulation during the rupture and formation cycles. Each row corresponds to a different molecule: benzene (top), cyclohexane (middle), and toluene (bottom).

### STM-Break Junction Experiments

Following the procedure detailed by de Ara et al.,^19^ the molecular electronics experiments were performed by the STM-BJ approach under ambient conditions. As electrodes, we used two gold wires (0.5 mm in diameter and of 99.99% purity as supplied by Goodfellow^50^), whose cylindrical surfaces were faced against each other in a perpendicular configuration. The voltage applied in all the experiments was 100 mV. The current through our molecular junctions was converted into volts in three stages of the current–voltage amplifier with respective gains of 10^6^, 10^8^, and 10^9^ V/A, in order to be recorded by analogue to digital converter (ADC) for the data acquisition (DAQ) system. This homemade instrument was tested and used in a previous publication,^19^ and it allows to record conductance in the range of *G*_0_ to 10^–5^*G*_0_.

Knowing the applied bias voltage and current, we can easily calculate the conductance, which is expressed in quantum of conductance (*G*_0_ = 2*e*^2^/*h*), where the factor of 2 comes from the spin degeneracy, *e* is the charge of the electron, and *h* is the Planck’s constant. Historically, the representation of conductance versus the relative displacement of the electrodes has been called a “trace of conductance.” These traces can be labeled as “rupture” or “formation,” depending on whether the electrodes are pulled apart or pushed together, respectively. In this article, we have only studied the rupture traces. As an internal protocol, we always use ultraclean gold electrodes, and we verify the extent of “cleanness” via a logarithmic histogram, which for clean electrodes is characterized by the nonexistence of any peak between the atomic contact (≈1 *G*_0_) and the floor noise of the *I*–*V* converter (≈10^–5^*G*_0_). Only after obtaining ultraclean gold, the organic solvent is deposited via drop casting over electrodes.^19^

## Results and Discussion

In order to gain an understanding of how the conductance is affected by the orientation of the molecule in relation to the connecting terminals and relative displacement between the electrodes, we have adopted a specific approach. In this approach, we keep the lower electrode and the molecular orientation (whether parallel or perpendicular) fixed, allowing only the displacement of the upper electrode in the direction indicated in Figure 1. We have studied the evolution of the conductance vs the relative displacement of the upper electrode for benzene, cyclohexane, and toluene molecules in the parallel and perpendicular configurations (see Figure 2). In all the cases, we have moved the upper electrode in step intervals of 0.1 Å without relaxation. At every step, we calculated the electronic transport (conductance) by DFT + NEGF. In Figure 2, the three panels show the conductance in units of *G*_0_ versus displacement up to 1.5 or 2.1 Å (depending on the molecule). This range has been selected to exclude the tunneling regime (1.6 to 5.0 Å for benzene and toluene and 2.2 to 5.0 Å for cyclohexane) due to the lack of information on interest (full range and details are displayed in Figure S1).

Figure 2 shows the evolution of the conductance for benzene, cyclohexane, and toluene. In each panel, the spheres represent parallel configurations, while triangles represent perpendicular configurations. The lines connecting the data points guide the eye. Moreover, the color code used in Figure 1 for every molecule also carries over to these panels a, b, and c. In direct correspondence with Figure 1, the “parallel” and “perpendicular” structures are indicated by blue- and gray-shaded areas, respectively. The height of this rectangle indicates the maximum and minimum values attributable to each configuration. This color code of blue and gray facilitates understanding of the experimental data.

From Figure 2, the first observation is that “parallel” configurations of benzene and toluene exhibit similar conductance traces during stretching. Furthermore, the calculated conductance traces for the perpendicular cases of benzene and toluene “perpendicular-L.R.” show the same behavior. These results suggest that the electronic transport in these aromatic molecules is relatively similar. In panel b, corresponding to cyclohexane (aliphatic), a subtle difference is observed where its range of relative displacement extends up to 2.1 Å (see Figure S2 for more details). Unlike aromatic molecules, the parallel configuration of cyclohexane exhibits a lower range of conductance values. However, in its perpendicular configuration, the conductance window of cyclohexane is seen to be similar to that of benzene in the range of 1.5 Å. These “toy-model” conductance’s calculations of idealized structures by DFT + NEGF are not representative of the vast quantity of possible positions that can be adopted by the molecule during the experiment. For this reason, we have decided to simulate via CMD simulations the different types of contacts that can likely be produced.

Figure 3 shows a summary of the snapshot of the cycles of rupture and formation obtained by CMD simulations. Each row corresponds to a different molecule, as indicated by the color code (black, purple, and green for benzene, cyclohexane, and toluene, respectively). At the top of each snapshot, we indicate the simulation cycle and step in units of kilosteps (ksteps, where 1 step equals 1 fs). The first column depicts the initial input structure used. For each molecule, multiple junction rupture and formation cycles are repeated. Representative snapshots are taken from different cycles at various steps in the cycle, illustrating the different positions that the molecules can take.

We analyzed only the rupture cycles, following the criterion of studying the breaking process of the experiments. In particular, we focused on the last point immediately before rupture, which enabled us to create the following classification hierarchy.

**Figure 4:**
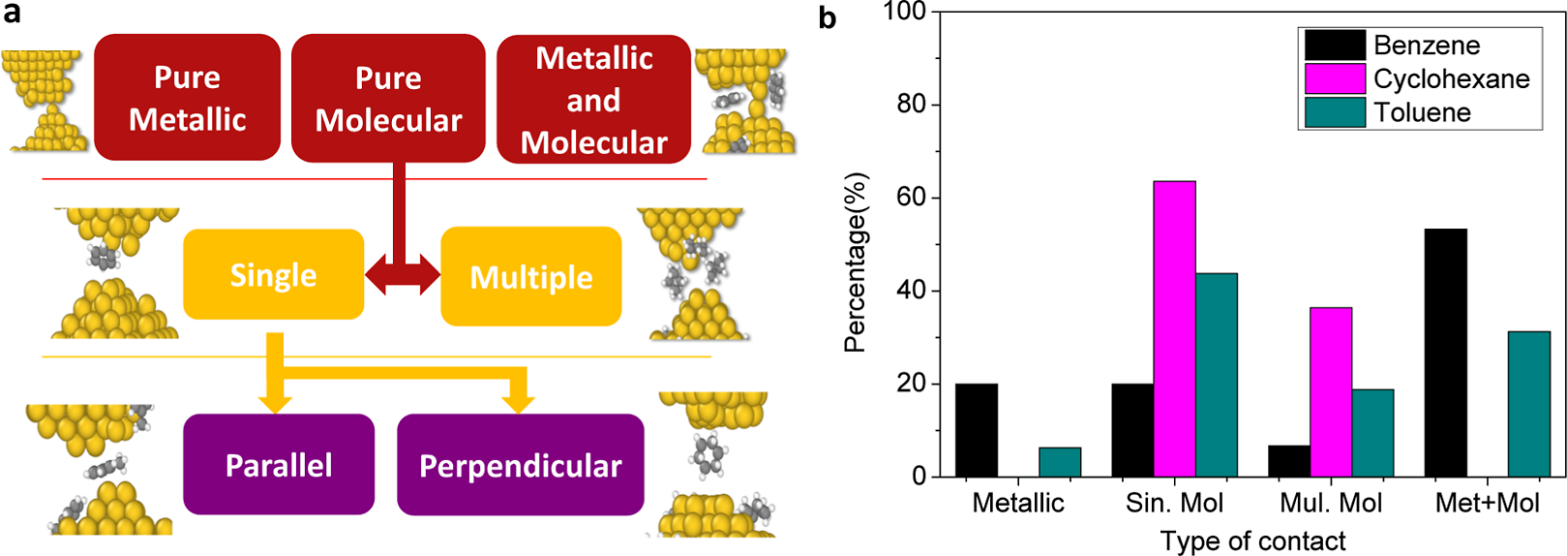
Panel (a) classification hierarchy of molecular contacts used in this work. To the left and right, we show example structures that illustrate each type of last contact. Panel (b) shows the percentage calculated from Table 1 for the different types of last rupture with benzene, cyclohexane, and toluene.

At the top of this hierarchy, the last contact before rupture can be purely metallic, purely molecular, or a combination of both. The red blocks shown at the top in Figure 4a represent these three common types of final contacts. The structures on the left and right of the red blocks depict final contacts composed of pure metal and a mixture of metal and molecules, respectively. When the final contact is purely molecular, two possibilities can occur: either a single molecule or multiple molecules are held by the nanojunction. This secondary classification of pure molecular contact is shown in the second row of Figure 4, and it is represented by yellow blocks in the diagram. Moreover, the structures to the left and right of these yellow blocks illustrate single or multiple molecular junctions, respectively. As Figure 1 illustrates, a single molecule can be captured in either a “parallel” or a “perpendicular” configuration by the junction, as indicated by the purple blocks in the final and lowest level of the classification hierarchy. The structure on the left of this last row illustrates the parallel orientation, while that on the right corresponds to the perpendicular one.

Thanks to our classification, we can determine how many times a given type of last molecular contact occurs in our simulations (Table 1). The first column indicates the molecule used in the simulation. The second column corresponds to the total number of rupture cycles analyzed. The third column represents the number of events in which we found a purely metallic contact at the moment of the last contact. The fourth column is the number of events that we have identified as the last contact involving a molecular contact (independent of whether the contacts contain single or multiple molecules). Finally, the fifth column shows the number of events in which we observed combined molecular and metallic contacts.

**Table 1:** Classification of the Last Type of Contact during the Simulated Process of the Rupture

molecule	cycles	metallic	molecular	Met + Mol
benzene	15	3	4	8
cyclohexane	11	0	11	0
toluene	16	1	10	5

Figure 4b represents in a bar graph the percentage of the events obtained in Table 1; the color code used is the same as in previous plots. From this figure, we observe clearly that the most probable last contact for cyclohexane and toluene is the single-molecule junction, and for the case of benzene, it is the combination of the metallic-molecular junction. However, from the results shown in Table 1 and Figure 4b, it is difficult to deduce a refined classification of the type of pure molecular junction that is possible. For this reason, we present Table 2 where the first column indicates the molecule, the second and third columns correspond to the single parallel and single perpendicular configurations, respectively, and the fourth column corresponds to the multiple molecular junctions that can be found at the moment of the last contact.

**Table 2:** Refined Classification of the Pure Molecular Junctions of Our Simulation Results^a^

molecule	Sin. Para.	Sin. Perp.	Mul. Mol.
benzene	2	1	1
cyclohexane	0	7	4
toluene	3	3	4

a The number of events of single parallel (Sin. Para.), single perpendicular (Sin. Perp.), and multiple molecule (Mul. Mol.) junctions

**Figure 5:**
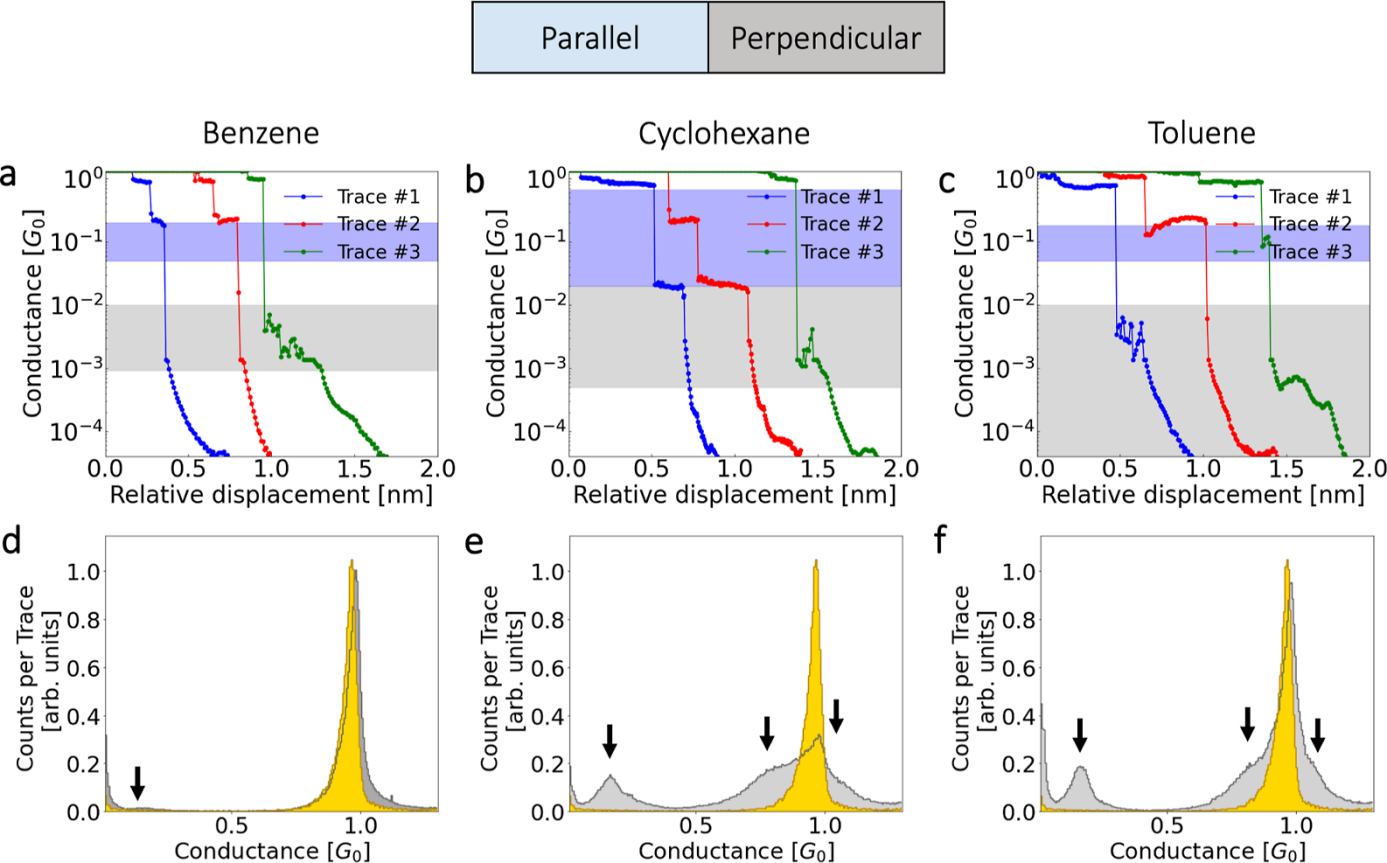
In panels (a–c), we show rupture traces for benzene, cyclohexane, and toluene, respectively. Gray- and blue-shaded areas represent the range of values of the theoretical traces calculated via DFT based on the different geometric configurations tested. Bottom panels (d–f) show one-dimensional experimental histograms of conductance for benzene, cyclohexane, and toluene. These histograms have been normalized with respect to the number of traces that contributed to each histogram bin, i.e., each bin count is divided by the total number of traces.

In order to ascertain the accuracy and reliability of our simulations and conductance calculations, we compared the simulation results with the experimental data. In each experiment, we first study bare gold, for which we usually collect over 2000 trace files as reference. Once we have finished the analysis of pure gold, we drop-cast the molecules over the bare gold and start the process of breaking and reforming of the junction, while at the same time collecting up to 10,000 traces of conductance. Typically, molecular signatures are observed under the 1 *G*_0_ region, so a logarithmic scale is usually most appropriate for analyzing the data statistically. A representative collection of experimental single rupture traces as a function of junction stretching is shown in Figure 5a–c. Each trace exhibits a different initial plateau-like behavior, followed by a plateau at lower conductance values or in the tunneling regime, related to the bridging of the electrodes by the molecules. Moreover, here we also display within the plots the blue and gray shading obtained from the DFT calculations shown in Figure 2. These shadings help us identify whether the plateau aligns with parallel or perpendicular configurations.

When we plot conductance histograms on the linear scale, we also observe humps or shoulders around the conductance of the metallic contact (1 *G*_0_). Figure 5 panels d–f show the experimental histograms for each molecule in light gray and for bare gold in gold color. The signals around the 1 *G*_0_ conductance can be ascribed to two different scenarios. The first could be a parallel configuration of the molecules compressed between the electrodes, so the coupling may be stronger and electrons hopping between the electrodes are also allowed, and hence the measured conductances are higher. The second scenario can be ascribed to the measurements of the gold atom with the molecule alongside it, providing greater stability to the junction. The data in Table 1 and Figure 4b show that the latter is the most likely scenario.

**Figure 6:**
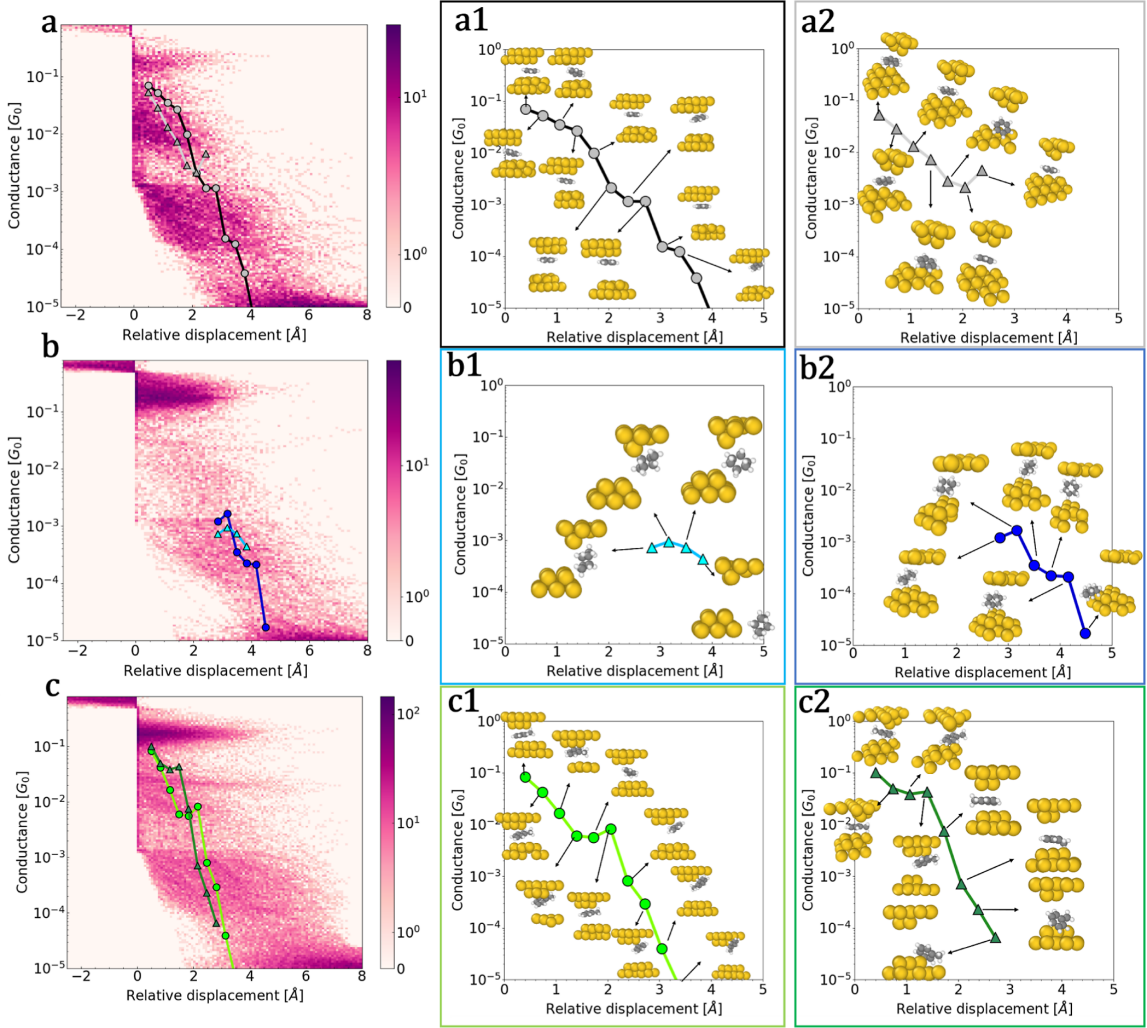
Experimental conductance–displacement density plots for benzene, cyclohexane, and toluene are shown in panels (a–c), respectively. The points connected by lines within these density plots represent ab initio transport values calculated for molecular junctions simulated through molecular dynamics. Additionally, the panels labeled as (a1,a2), (b1,b2), and (c1,c2) correspond to benzene, cyclohexane, and toluene. In each panel, the calculated conductance versus relative displacements are displayed, overlaid on the density plots, along with illustrations corresponding to each point, allowing for a reference of the molecular contact geometry. The color code for the theoretical data and overlaid on the experimental data corresponds to that used in the side panels, where the colors of the box and the trace are the same.

In Figure 2, idealized scenarios are shown, the results of which are useful to get a global picture of the role of the molecular orientation on conductance but are not able to accurately reproduce the dynamic processes in our experiments. We propose a dynamic and informative representation of the possible binding configurations that a molecule can adopt when stretched between two gold electrodes. To achieve this, we present Figure 6, which includes experimental data for three different molecules (benzene, cyclohexane, and toluene) shown as 2D histograms (density plots). Additionally, we compare these experimental results with two selected scenarios of molecular junctions obtained from the CMD simulations. For these scenarios, we have performed electronic transport calculations via DFT, as shown in insets a1, a2, b1, b2, c1, and c2. Within these figures, the relative displacement can be adjusted or offset. To reduce computational time in DFT calculations, we focused on molecular junctions by reducing the initial number of atoms in the electrodes. The resulting molecular junctions are illustrated in panels a1–c2. These illustrations require the addition of three layers of atoms in the upper and lower parts of the top and bottom electrodes. Overall, Figure 6 provides a comprehensive view of the binding configurations of the studied molecules and enables a direct comparison between experimental data and theoretical simulations. For constructing the experimental 2D histograms, we considered traces that present molecular junctions under 0.7 *G*_0_ and aligned them to the value of 0.5 *G*_0_ to enhance solely the molecular contribution. The color maps on the logarithmic scale show the most repeated values. Overlaid on the 2D histograms are the computed conductance values by DFT of molecular junctions simulated through molecular dynamics. These overlaid traces on the density maps are also presented in separate adjacent panels. These panels, in turn, display the traces calculated using our combination of MD and DFT, along with illustrations showing how the simulated contact evolves when stretching the electrodes. The borders of the rectangles in these panels are colored according to the theoretical trace. In panel a1, it is evident that the benzene molecule initially remains parallel for the first displacements. However, once it exceeds 1 Å, it reorients itself perpendicularly and eventually attaches flatly to one of the electrodes. This leads to a plateau in the range of 10^–3^ to 10^–4^*G*_0_ during the stretching process. As the displacement reaches approximately 3.5 Å, the molecule no longer seems to be bound to the lower electrode, as it is displaced upward together with the upper electrode, and its conductance begins to decline.

In panel a2, we observe that the molecule starts in a parallel position. However, shortly before reaching a displacement of 1 Å, the molecule transitions to a perpendicular orientation, exhibiting conductance values between 10^–2^ and 10^–3^*G*_0_. Based on these theoretical findings, we can extrapolate that for benzene, values close to 10^–1^*G*_0_ correspond to the parallel configuration, while conductance values below 10^–1^, approaching 10^–3^*G*_0_, indicate slightly perpendicular configurations. Comparing computational results with experimental data suggests that when values fall within the range of 10^–3^ to 10^–5^*G*_0_, it is likely that the molecule has become anchored to one of the electrodes.

Moving on to the cases of cyclohexane represented by panels b1 and b2, we observe that in both cases, the molecule starts in a perpendicular configuration. In both panels, the conductance is in the range from 10^–3^ to 10^–4^*G*_0_, with a relative displacement starting at approximately 2.5 Å and finishing around 4.0 Å. Therefore, when comparing these observations with experimental data, it is plausible to associate values ranging from 10^–3^ to 10^–4^*G*_0_ with this specific geometric arrangement.

Finally, we have toluene, which exhibits a behavior very similar to the other aromatic molecule. In panel c1, we observe that it initially assumes a parallel configuration with a conductance in the range of 10^–1^*G*_0_. Once the displacement exceeds 1 Å, the molecule gradually orients itself slightly toward a perpendicular position, resulting in a conductance plateau of approximately 10^–2^*G*_0_ for an approximate distance of 1 Å. Eventually, the molecule becomes detached from one of the electrodes, and its conductance takes on tunneling characteristics.

Similarly, in panel c2, we start with a molecule in a parallel position that remains stable until it reaches almost 1.4 Å. Subsequently, the molecule becomes unanchored, and its conductance exponentially decreases as would be expected for a tunneling conductance regime. In summary, for toluene, the experimental observations suggest that plateaus around 10^–1^*G*_0_ could be associated with parallel geometries, while the plateau close to 10^–2^*G*_0_ might indicate slightly perpendicular configurations.

The three panels a, b, and c of Figure 6 provide insight into the detailed molecular orientations of the molecules in the junction during the junction break process, with good agreement between experimental data and the results obtained from our theoretical model consisting of CMD and DFT + NEGF.

To summarize, the experimental observations in aromatic molecules indicate that plateaus near 10^–1^*G*_0_ can be associated with parallel geometries and the plateau close to 10^–2^*G*_0_ suggests slightly perpendicular configurations. Low conductances on the order of 10^–3^ to 10^–5^*G*_0_ correspond to the molecule losing contact with one of the electrodes and becoming anchored on the other, with conductivity exhibiting tunneling characteristics. For aliphatic molecules, conductance values around 10^–3^ to 10^–4^*G*_0_ correspond to perpendicular configuration. These findings provide valuable insights into the conductance behavior of these aromatic and aliphatic molecules and contribute to our understanding of their electronic properties in nanoscale systems.

## Conclusions

Through a combination of electronic transport experiments under ambient conditions, molecular dynamics simulations, and DFT transport calculations of the conductance, we have untangled the relation between the electrical conductance and binding conformations of aromatic and aliphatic molecular junctions.

The DFT-based transport calculations that were performed on idealized structures reproduce the range of conductance values observed experimentally concerning the stretching of benzene, cyclohexane, and toluene molecular junctions. Subsequently, CMD simulations aided in the identification and classification of the last contact, which is likely formed immediately before rupture in the experiments. Moreover, CMD simulations generated different scenarios of the contacts that allowed us to compute the electronic transport and obtain statistics, enabling us to compare them with the experimental data. Thanks to the broad agreement between the results from MD and DFT, on the one hand, and the experimental conductance clouds in the density plots, on the other hand, we can at least elucidate the geometric configurations that these molecules adopt in the experiments. We further believe that the classification that we have established for the different types of molecular contacts can be applied in molecular electronics involving all manners of ring-shaped or planar molecules.

The combination of our previous findings^19^ and the results presented in this article reveal that the solvents benzene, cyclohexane, and toluene are never fully evaporated and remain adsorbed on the electrodes. Our simulations, calculations, and experiments thus allow us to obtain the characteristic conductance values of these solvents, such that when they are employed alongside other target molecules in future experiments, we will be able to clearly distinguish the conductance signatures of the solvents and their geometric relationships with the electrodes. Thanks to this study, when analyzing the conductance using break-junction methods for any molecule dissolved in one of these three solvents, we will be able to understand the role played by the solvent in the measurement.

## Supporting Information Available

The evolution of the calculated conductance versus the relative displacement in the range of 0 Å to 6 Å carried out through DFT calculations with the HSE06 functional; evaluation of the three functionals: BLYP, B3LYP, and HSE06; complementary experimental analysis involving the use of 2D histograms for clean gold; and the comparison of 2D histograms with accepted and rejected traces for the solvents benzene, toluene, and cyclohexane (PDF)
