# Supplementary material for: Unraveling the Interplay between Quantum Transport and Geometrical Conformations in Monocyclic Hydrocarbons’ Molecular Junctions
Source: J Phys Chem C Nanomater Interfaces. 2023 Nov 27;127(48):23303–11. doi: 10.1021/acs.jpcc.3c05393 (PMC10861133; doi:10.1021/acs.jpcc.3c05393)
Supplement: Supplementary file 1 — jp3c05393_si_001.pdf [file jp3c05393_si_001.pdf]

Unraveling the Interplay between Quantum Transport and Geometrical Conformations in Monocyclic Hydrocarbons Molecular Junctions

A. Martinez-Garcia,† T. de Ara,† L. Pastor-Amat,† C. Untiedt,† E. B. Lombardi,‡ W. Dednam,‡ and C. Sabater*,†

†*Departamento de Física Aplicada and Instituto Universitario de Materiales de Alicante (IUMA), Universidad de Alicante, Campus de San Vicente del Raspeig, E-03690 Alicante, Spain.*

‡*Department of Physics, Florida Science Campus, University of South Africa, Florida Park, Johannesburg 1710, South Africa*

E-mail: carlos.sabater@ua.es

Supplementary information

DFT calculations based on HSE06

Fig.S1 shows the evolution of the calculated conductance vs. the relative displacement in the range 0 Å to 6 Å, in other words where the tunnelling regime can be noticed. In all the cases, we have separated the upper electrode in step intervals of 0.1 Å without relaxation and we have calculated the conductance by DFT+NEGF. The three panels show the conductance in units of G_0 for benzene, cyclohexane and toluene molecules in parallel and perpendicular configurations. The red lines indicate the slope of the tunnelling regime. The yellow strip highlights the first value that starts to deviate from the tunnelling regime, as indicated by the red trend line. We utilized the highlighted relative displacement value to create Fig.2.

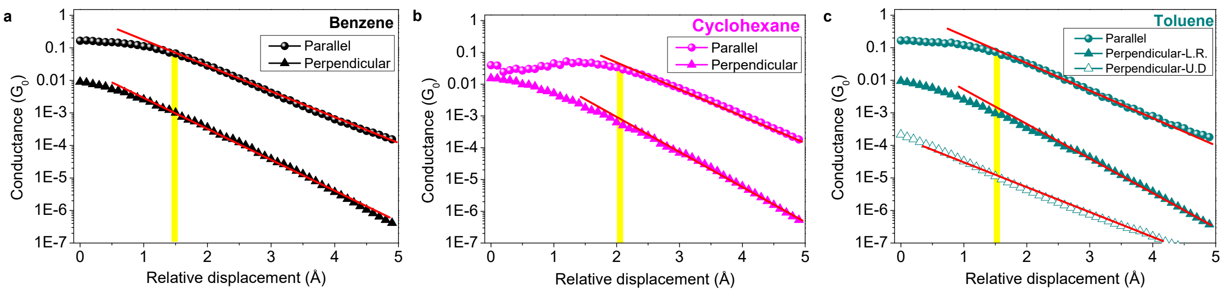

Figure S1: Electronic transport vs relative displacement calculated for the initial structures in panel (a) benzene, (b) cyclohexane and (c) toluene. Coloured markers represent the calculated values of gold (orange), benzene (black), cyclohexane (pink) and toluene (green).

Comparison between functionals

In this section, we will compare the conductance versus displacement for all three molecules in their parallel and perpendicular positions. We will also compare the three functionals: BLYP, B3LYP, and HSE06.

In light of the results shown in Fig.S2, the HSE06 and B3LYP functionals show very similar outcomes, with the difference being that B3LYP is computationally more affordable.

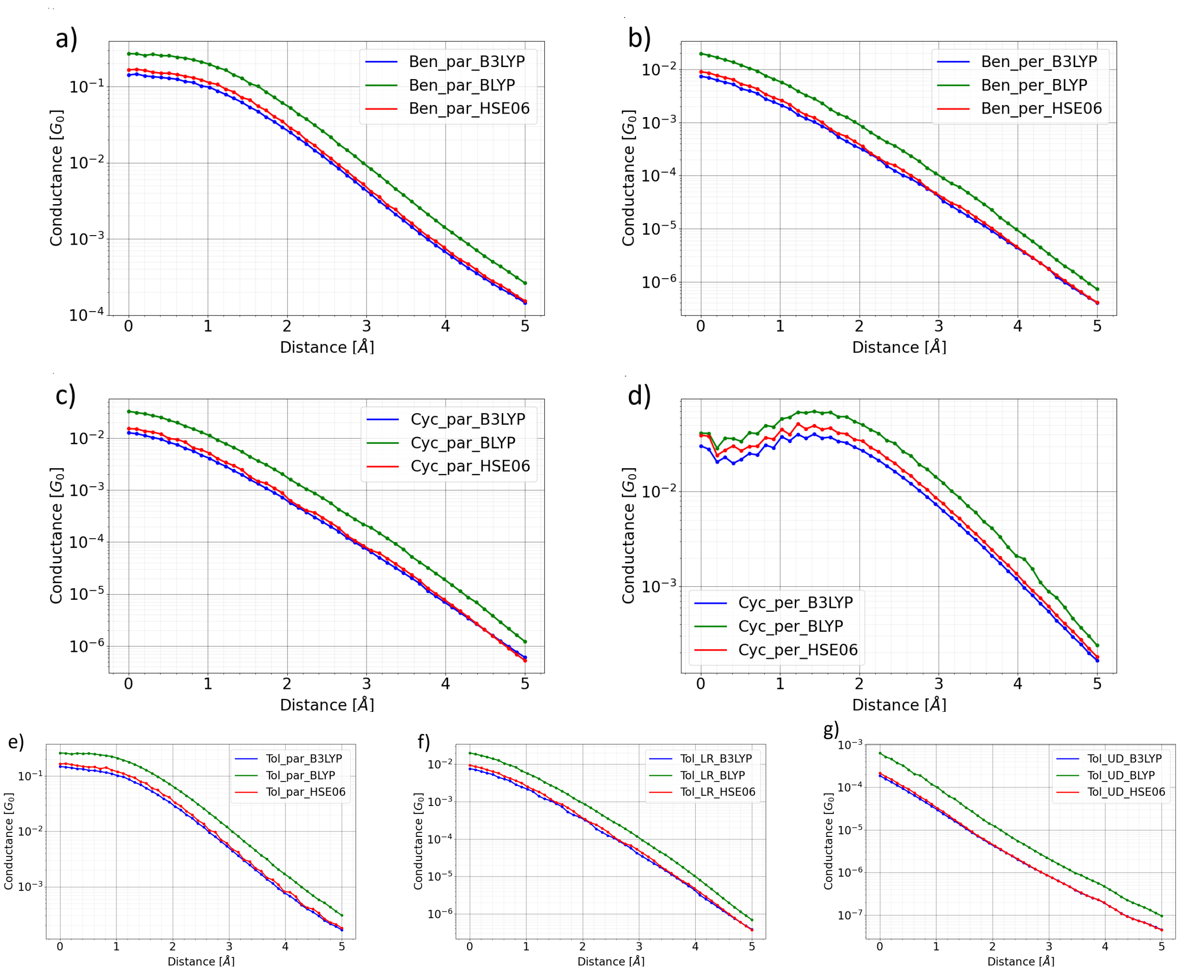

Figure S2: The green, blue, and red lines represent the calculated values for the BLYP, B3LYP, and HSE06 functionals, respectively. Panels a) and b) correspond to benzene in its parallel and perpendicular configurations, respectively. Panels c) and d) show cyclohexane in its parallel and perpendicular configurations, respectively. Panel e) represents toluene in its parallel configuration, while panels f) and g) show toluene in its perpendicular configuration with L.R. and U.D. orientations, respectively.

However, as expected, we do observe that the BLYP potential, optimized exclusively for metals, yields different results compared to the hybrid functionals.

It is important to note that the BLYP functional is primarily designed for metallic systems and may not accurately capture the electronic properties of non-metallic molecules. Hybrid functionals like HSE06 and B3LYP incorporate a combination of Hartree-Fock exchange and density functional theory, allowing for a more comprehensive description of electronic behaviour in various systems, including both metals and non-metals. Furthermore, the

computational affordability of B3LYP makes it an attractive option for larger-scale studies or those with limited resources.

Despite variations arising from different functionals, it's worth noting that the difference is still lower than the experimental variability in the conductance of the diverse molecular configurations.

2D Histograms complementary analysis

As a reference, in Fig. S3 is shown a 2D histogram of clean gold on a semi-log scale. All the traces were taken using a bias voltage of 0.1 V. Here, the alignment point of the traces is 0.5 G_0, following the alignment point of the 2D histograms presented for each organic molecule. As we can observe, statistically, no significant structures occur below 1 G_0. The main contribution arises from tunnelling events, which initiate at values below 10^{-2} G_0.

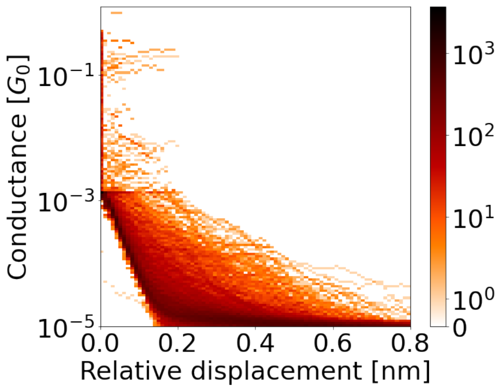

Figure S3: 2D histogram of clean gold.

Additionally, Fig. S4 presents the 2D histograms of the rejected traces in panels (a1), (b1) and (c1) along the admitted traces (a2), (b2) and (c2) for comparison purposes. Here we observe the traces with long molecular plateaus which are more significant and have been included in the analysis in the main manuscript. Differences among the three organic molecules are observed which support the statement we are measuring the target molecules and not contaminants.

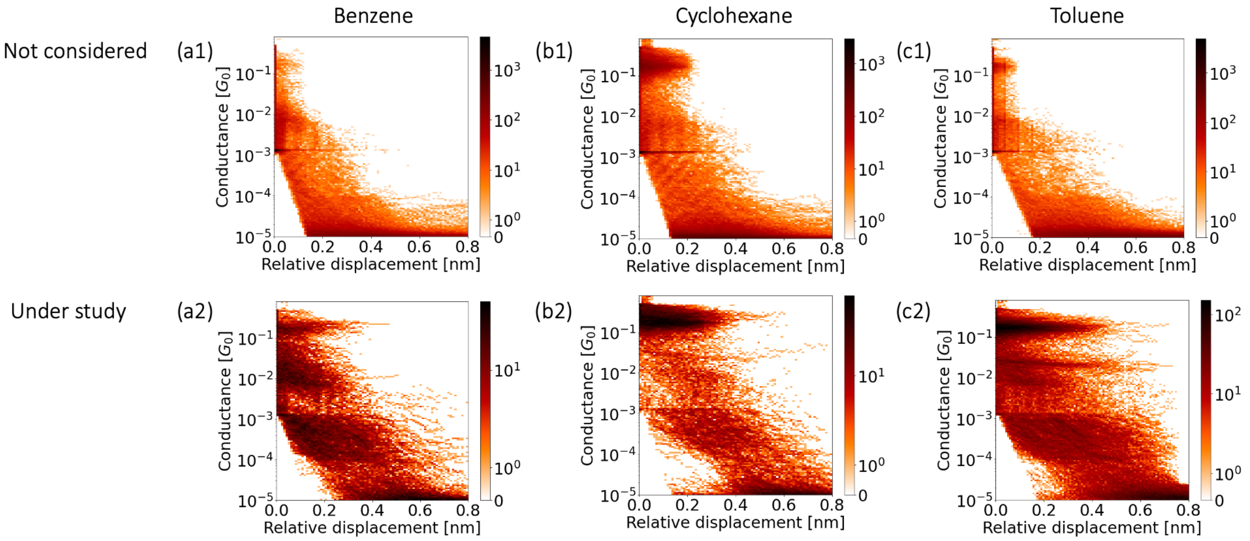

Figure S4: 2D histograms of the data set measured during the experiments for (a) benzene, (b) cyclohexane and (c) toluene at 100 mV. The first row of the figure shows the discarded traces for the analysis, whereas the second row presents the histograms of the data with relevant molecular plateaus. Although discarded traces still present molecular behaviour, the events themselves are few with short plateau lengths.
